# Anti-atherosclerotic effect of *Cynodon dactylon* extract on experimentally induced hypercholesterolemia in rats 

**Published:** 2017-09-15

**Authors:** Belal Pashaie, Rahim Hobbenaghi, Hassan Malekinejad

**Affiliations:** 1 Department of Pathobiology, Faculty of Veterinary Medicine, Urmia University, Urmia, Iran;; 2 Department of Basic Sciences, Faculty of Veterinary Medicine, Urmia University, Urmia, Iran;; 3 Department of Pharmacology and Toxicology, Faculty of Pharmacy, Urmia University of Medical Sciences, Urmia, Iran.

**Keywords:** Atherosclerosis, *Cynodon dactylon*, Hypercholesterolemia, Lipid profile

## Abstract

*Cynodon dactylon* (Bermuda grass) is a perennial plant traditionally used as an herbal medicine in many countries. In the present study, anti-atherosclerotic property of ethanolic extract of *C. dactylon* was investigated in the experimentally induced hypercholesterolemia in rats. In this study, 36 male Wistar rats were selected and allocated into six groups (n = 6). The control group received a normal diet, sham group received a high cholesterol diet (HCD; 1.50% cholesterol and 24.00% fat) and other groups received a HCD and ethanolic extract of *C. dactylon* at low (100 mg kg^-1^), moderate (200 mg kg^-1^) and maximum (400 mg kg^-1^) doses via gavages. The last group received atorvastatin (10 mg kg^-1^) through gavage with a HCD. The study period for all groups was six months. At the end of this period, parameters including total cholesterol (TC), triglyceride (TG), low-density lipoprotein cholesterol (LDL-C) and high-density lipoprotein cholesterol (HDL-C) were assessed in the blood samples. Additionally, histopathological and immunohistochemical examinations on coronary and aorta arteries sections were performed. The results showed an increase in vessels wall thickness and proliferation of smooth muscle cells in the HCD group, while these pathological changes were not seen in *C. dactylon*-treated groups. Treatment of HCD animals with *C. dactylon* positively changed lipid profile by lowering of TC, TG and LDL-C. The results indicate that *C. dactylon* prevents from early atherosclerotic changes in the vessels wall.

## Introduction

Atherosclerosis is one of the major pathological conditions for cardiovascular disease, characterized by lipid and cholesterol deposition on the inner lining of arteries, resulting in plaque formation, narrowing of the lumen and eventually blood flow turbulence.^1^ There are several risk factors for atherosclerosis including abnormal lipid metabolism, inflammatory reactions and dysfunction of endothelial lining of arteries.^2^ The association between plasma lipid levels and risk of atherosclerosis has been reported.^3^^,^^4^ Dysfunctions of endothelial lining of lesion-prone areas of the arterial vasculature are early markers for atherosclerosis.^5^ Atherosclerotic plaque should be considered as an inflammatory response to injury.^6^ An increase in blood concentration of apolipoprotein B containing of low-density lipoprotein (LDL) is the most prevalent cause of atherosclerosis.^7^ The abnormal attachment of LDL to intimal proteoglycans is an important step for disease initiation which may potentially explain the atherosclerosis proneness of adaptive intimal thickenings. There are also reports indicating atherosclerosis development even at lower levels of LDL in combination with other risk factors facilitating atherosclerosis (multifactorial disease). These risk factors include smoking, hypertension, diabetes mellitus, male sex and a complex genetic susceptibility to the disease.^8^ Monocytes, macrophages and macrophage-derived foam cells exert manifold effects on lesion development and participate in the processes mediating the progression of atherosclerotic plaque composed of lipid, secretion of pro-inflammatory and cytotoxic factors and extracellular matrix (ECM) remodeling.^9^^,^^10^

Atorvastatin (STN) is routinely prescribed as a therapeutic agent for atherosclerosis because of its blood lipid-lowering, anti-inflammatory and endothelial protective activities.^11^ Statins inhibit 3-hydroxy-3-methylglutarylcoenzyme A reductase competitively, reduce LDL levels and lower triglyceride levels in hypertriglyceridemic patients.^12^ Statins anti-atherosclerotic effects correlate positively with LDL cholesterol reduction and are independent of their hypolipidemic action; nevertheless statins have side effects such as liver and muscle toxicities.^13^

*Cynodon dactylon* (Bermuda grass) is a perennial plant found in all over the world and particularly is native to the warm temperate and tropical regions.^14^ The plant is traditionally used as an agent to control diabetes in India and the extract of *C. dactylon* leaf has been declared to have antidiabetic, antioxidant, hypolipidemic and immunomodulatory effects.^14^ *Cynodon dactylon *has potent 2, 2-diphenyl-1-picrylhydrazyl free radical and nitric oxide scavenging activities.^15^^,^^16^ The plant is rich in metabolites remarkably proteins, carbohydrates, minerals, flavonoids, carotenoids, alkaloids, glycosides and triterpenoides.^17^ Whole plant of *C. dactylon* shows several biological activities such as antimicrobial, antiviral and wound healing properties.^17^ Important phyto-constituents reported from this plant were flavonoids including apigenin, luteolin, orientin and vitexin.^18^ Flavonoids may play a major role as they have been proven as anti-inflammatory agents due to their inhibitory effects on enzymes involved in production of inflammatory mediators.^19^ There are studies indicating that *C. dactylon* possess similar capabilities as STN does, thereby making it a candidate for considering in atherosclerosis therapy.^20^ Therefore, the aim of this study was to evaluate the cholesterol lowering and anti-atherogenic properties of *C. dactylon*in compared to STN in diet-induced hypercholesterolemia in rats.

## Materials and Methods

**Animals.** Adult healthy male Wistar rats weighing about 200 to 250 g were obtained from Urmia University, Department of Basic Sciences and used in this study. All of the animals were fed with a pellet diet (standard commercial diet) and water was provided *ad libitum*. Prior to experiments, the rats were acclimatized for a period of 7 days in standard environmental conditions including temperature (24 ± 2 ˚C), relative humidity (45 to 55%) and 12/12 hr dark/light cycle. The experiments were performed on animals in accordance with the guidelines of the ethical committee for research on laboratory animals of Urmia University.

**Hypercholestrolemia induction.** The 24 weeks old male rats were fed with a high cholesterol diet (HCD) for six months.^21^^,^^22^ Hypercholestrolemia was induced as described previously, with the addition of dried egg yolk (50.00%) to the whole feed and providing diet with 1.50% cholesterol and 24.00% fat.^23^ Also, 0.20% propylthiouracil (PTU) was added to the daily diet in order to decrease the amount of thyroid hormone produced by thyroid gland and block the conversion of thyroxineto T3.^24^ Thyroid hormone directly promotes cholesterol metabolism by the liver and PTU induces high blood levels of cholesterol.^24^^,^^25^ The rats in the control group were fed with standard laboratory diet.

**Preparation of** ***C. dactylon*** **extract.** *C. dactylon* rhizomes were collected from the suburbs of Urmia (West Azerbaijan, Iran) identified and authenticated by a plant taxonomist in Urmia University, Faculty of Agriculture. The plants were cleaned and dried at room temperature for 10 days and coarsely powdered. Extraction was performed with 20 g of powdered plant material and 200 mL 70% ethanol in a soxhlet extractor at 45 to 50 ˚C. The extraction was continued until the solvent in the thimble became clear indicating the completion of extraction. After each extraction, the solvent was distilled under vacuum below 50 ˚C using a rotary evaporator. The yield was 11.66% (w/w). Such dried extracts were stored in the refrigerator until using for bioassay tests.

**Experimental design.** Before the experimental procedures, rats were randomly divided into control and test groups (n = 6) as follows:

Group I (Control): the animals in this group were served as control and received normal saline (1 mL per rat) via gastric tubes for six months; Group II: the animals in this group were served as sham group and received hypercholesterolemic diet for six months; Group III: the animals in this group were received hypercholesrolemic diet and 100 mg kg^-1^ extract of *C. dactylon* via gastric tubes for six months; Group IV: the animals in this group were received hypercholesrolemic diet and 200 mg kg^-1^ extract of *C. dactylon *via gastric tubes for six months; Group V: the animals in this group were received hypercholesrolemic diet and 400 mg kg^-1^ extract of *C. dactylon* via gastric tubes for six months and Group VI: the animals were received hypercholesrolemic diet and STN (10 mg kg^-1^) via gastric tubes for six months.

**Serum preparation and tissue samples collections.** Following anesthesia with diethyl ether, blood samples were collected directly from the heart. The blood samples were centrifuged at 3000 *g* for 10 min to obtain sera which were stored at –20 ˚C for further analyses. Anesthetized animals were humanely euthanized using CO_2_ gas in a special device and immediately the heart and aorta were removed and rinsed with chilled normal saline. The samples were fixed in 10% phosphate buffered saline (PBS) formalin for pathological examinations.

**Lipid profile measurement.** Concentrations of total cholesterol (TC), total triglycerides (TG), high-density lipoprotein cholesterol (HDL-C) and low-density lipoprotein (LDL-C) in serum were determined by enzymatic colorimetric methods using commercial kits (Pars Azmoon, Tehran, Iran).

**Histopathological analysis.** The samples of heart and aorta were fixed in 10% paraformaldehyde for histological studies. The washed tissues were dehydrated in increasing gradient of ethanol and finally cleared in toluene. The tissues were then embedded in molten paraffin wax. Sections were cut at 5 µm thickness and stained with hematoxylin and eosin (H & E) for microscopical examinations. Heidenhain's Azan trichrome staining was performed to show connective tissue, especially muscle, collagen and nuclear chromatin in histological sections. This method was used to study the changes of aortic wall and coronary arteries.

**Immunohistochemical (IHC) analyses for proliferating cell nuclear antigen (PCNA).** The IHC staining was done in order to analyze the PCNA positive cell distribution. Before staining, the tissue sections (5 µm) were heated at 60 ˚C for approximately 25 min in a hot air oven (Venticell, MMM, Einrichtungen, Germany). The sections were then de-paraffinized in xylene (two changes) and rehydrated using alcohol gradients (96%, 90%, 80%, 70% and 50%). The antigen retrieval process was performed in 10 mM sodium citrate buffer (pH: 7.20). The IHC staining was conducted according to the manufacturer's protocol (Biocare Medical, Concord, USA). Briefly; endogenous peroxidase was blocked in a peroxidase blocking solution (0.03% hydrogen peroxide containing sodium acid) for 5 min. Tissue sections were then washed gently with PBS (pH: 7.0) and subsequently incubated with PCNA (1: 800) biotinylated primary antibodies (Biocare Medical) overnight at 4 ˚C in a humidified chamber. The sections were rinsed gently with PBS. After that, the slides were incubated with 50-100 μL of streptavidin–horseradish peroxidase (Sigma-Aldrich, Darmstadt, Germany) for 20 min. Subsequently, the tissue sections were rinsed gently in washing buffer and placed in a buffer bath. A DAB chromogen was added to the tissue sections and incubated for 10 min. Then, the slides were counter stained with hematoxylin for 10 sec. The sections were then dipped in weak ammonia (0.037 mL) 10 times, rinsed with distilled water and cover slipped.

**Statistical analysis.** Statistical differences were calculated using one-way analysis of variance (ANOVA) followed by Bonferroni post hoc test using Graph-Pad Prism version 6.07; GraphPad software Inc., San Diego, USA). For evaluating the pixel based frequency of IHC staining, the images were taken by on-board camera (Sony Corporation, Tokyo, Japan) and analyzed by Image pro-insight software (version 9.00; Cybermetric Co., Phoenix, USA). A *p*-value less than 0.05 was considered as statistically significant.

## Results

**Plasma level of cholesterol, LDL and HDL.** Feeding rats with high cholesterol supplemented diet resulted in a significant increase in plasma cholesterol, LDL and HDL levels (Table 1). The levels of cholesterol and LDL significantly (*p *< 0.05) reduced after administration of *C. dactylon *extract and STN in test groups. The extract reduced TC levels to 29.90%, 40.94% and 42.51% by 100 mg kg^-1^, 200 mg kg^-1^ and 400 mg kg^-1^ doses, respectively. Moreover, treatment with STN (10 mg kg^-1^) significantly (85.19%) reduced TC level (Table 1), (*p* < 0.05).

**Plasma level of TG.** The blood triglyceride levels were increased significantly in HCD group (Table 1) compared to control rats (*p *< 0.05). In treatment groups, blood triglyceride levels significantly decreased.

**Fig. 1:**
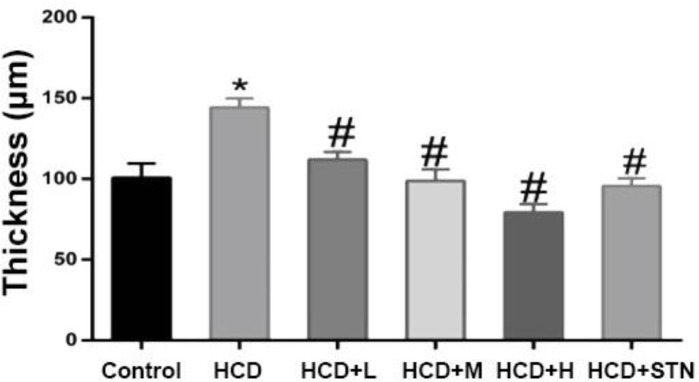
Measurements of intima and media layers thickness of thoracic aortas from rats treated with or without *C. dactylon* (n = 6).  *^,^^#^ indicate significant differences compared to control and non-treated HCD groups at *p* < 0.05, respectively. HCD: high cholesterol diet; HCD+L: low dose of *C. dactylon*; HCD+M: medium dose of *C. dactylon*; HCD+H: high dose of *C. dactylon* and HCD+STN: HCD+atorvastatin

**Fig. 2:**
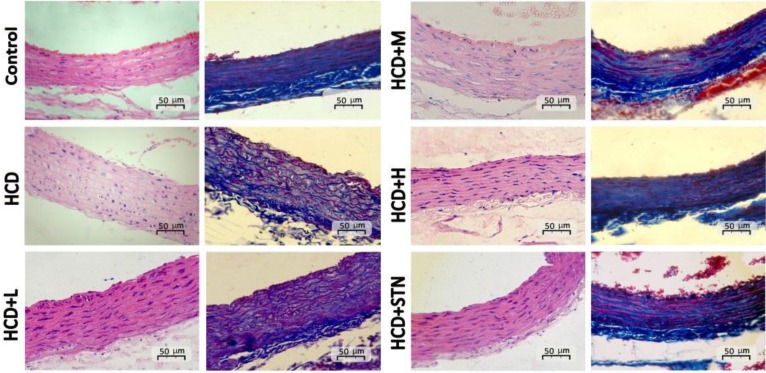
Photomicrographs of H & E (left) and Azan trichrome (right) cross sections of the thoracic aortas of control, high cholesterol diet (HCD), low dose (100 mg kg^-1^) of *C. dactylon* (HCD+L ), medium dose (200 mg kg^-1^) of *C. dactylon* (HCD+M ), high dose (400 mg kg^-1^) of *C. dactylon* (HCD+H) and atorvastatin (10 mg kg^-1^) group (HCD+STN

**Fig. 3:**
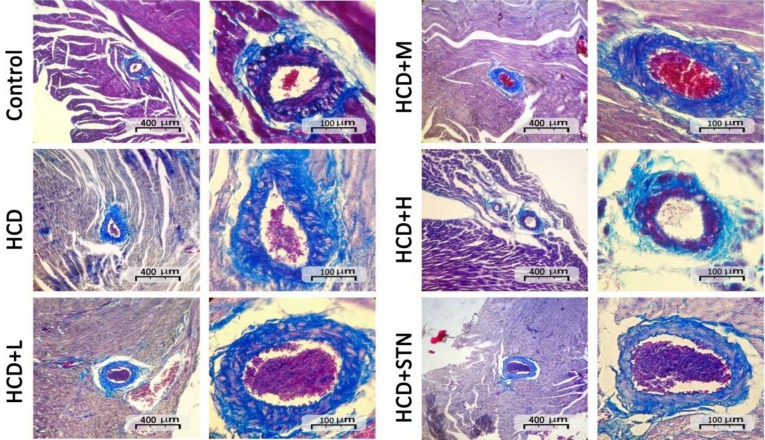
Photomicrographs of Azan trichrome cross sections of the coronary artery. Density of blue colored fibers indicates vascular wall fibrosis. HCD: high cholesterol diet; HCD+L: low dose of *C. dactylon*; HCD+M: medium dose of *C. dactylon*; HCD+H: high dose of *C. dactylon* and HCD+STN: HCD+atorvastatin

**Histopathological findings.** Figure 1 represents the intima and media thickness of thoracic aortas from different groups. As shown in Figure 2, the aortic intimal surface is smooth and intact in control group, while intimal and medial layers of aorta in HCD rats showed abnormalities, characterized by medial and internal elastic lamina thickness and irregularity. Also, there was an increase in aortic wall diameter after chronic consumption of HCD. These histopathological changes were not seen in treatment groups, as in high dose of the extract, histological appearance of aorta is very similar to control and STN groups. Cross sections of coronary artery wall from HCD group showed medial thickness and vascular fibrosis characterized by high density of blue color fiber appearance. In 400 mg kg^-1^ treated group, vascular fibrosis and blue density were lowered (Fig. 3). 

**Fig. 4:**
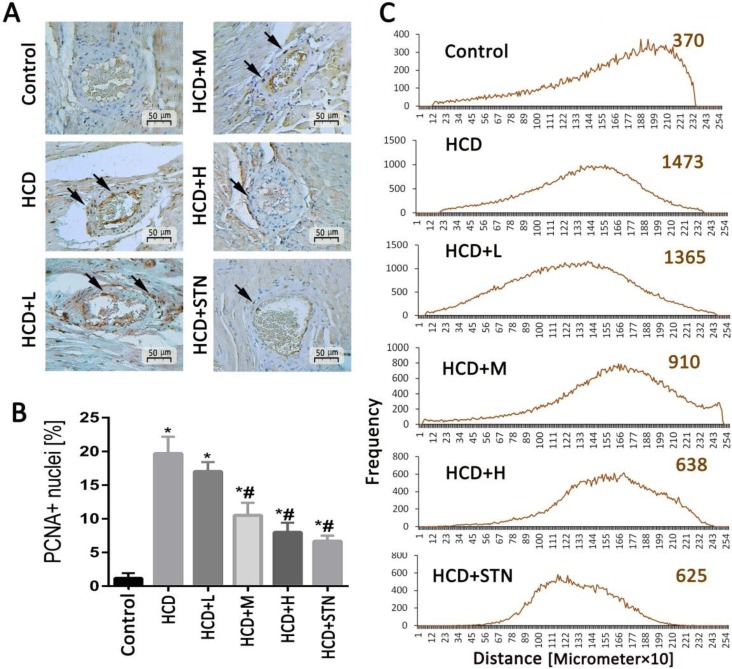
Analysis of cellular proliferation. A) Nuclear positivity for proliferating cell nuclear antigen (PCNA) immunostaining (arrows) increases within coronary artery regions indicate the association of observed vascular remodeling with smooth muscle cells proliferation, PCNA immunostaining,. B) Analysis of the density of PCNA-positive cells. C) Software analyses for distribution of PCNA positive cells (pixel-based histogram). Each value represents the average positive-cell density and the mean ± SD of six animals. *^,#^ indicate significant differences compared to control and non-treated HCD groups at *p* < 0.05, respectively. HCD: high cholesterol diet; HCD+L: low dose of *C. dactylon*; HCD+M: medium dose of *C. dactylon*; HCD+H: high dose of *C. dactylon* and HCD+STN: HCD+atorvastatin

**Fig. 5:**
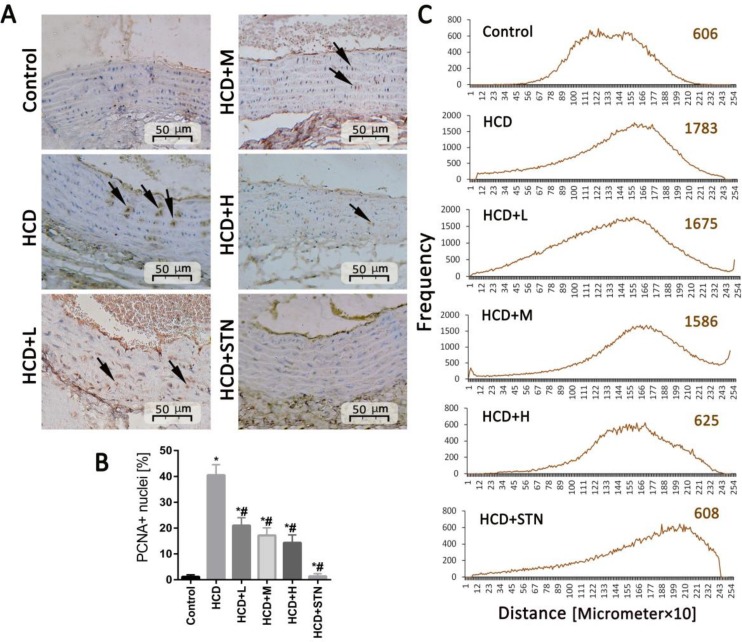
Proliferating cell nuclear antigen (PCNA) staining of aortic cross sections. A) Nuclear positivity for PCNA immunostaining (arrows) increases with in aorta wall indicate that observed vascular remodelling is associated with smooth muscle cells proliferation, PCNA immunostaining,. B) Analysis of the density of PCNA-positive cells. C) Software analyses for distribution of PCNA positive cells (pixel-based histogram). Each value represents the average positive-cell density and the mean ± SD of six animals. *^,#^ indicate significant differences compared to control and non-treated HCD groups at *p* < 0.05, respectively. HCD: high cholesterol diet; HCD+L: low dose of *C. dactylon*; HCD+M: medium dose of *C. dactylon*; HCD+H: high dose of *C. dactylon* and HCD+STN: HCD+atorvastatin

Immunostaining of coronary arteries and aorta walls (Figs. 4 and 5) with PCNA revealed a large amount of vascular smooth muscle cells (VSMC) proliferation in HCD group. However, the expressions of PCNA were reduced conspicuously in *C. dactylon*-administrated groups especially in high doses.

**Table 1:** Effects of ethanolic extract of *C. dactylon *on plasma lipid profile after six months in control and experimental animals (n = 6). Data are presented as means ± SD

**Parameters**	**Control**	**HCD**	**HCD+L**	**HCD+M**	**HCD+H**	**HCD+STN**
**Total cholesterols (mg dL** ^-1^ **)**	76.6 0 ± 4.45	127.00 ± 3.47^a^	89.00 ± 3.91^b^	75.40 ± 2.90^b^	73.60 ± 3.58^b^	18.80 ± 3.30^ab^
**HDL (mg dL** ^-1^ **)**	37.80 ± 2.22	50.40 ± 3.44^a^	36.60 ± 2.46^b^	42.20 ± 1.24	40.80 ± 1.46^b^	44.20 ± 1.59^a^
**LDL (mg dL** ^-1^ **)**	76.00 ± 2.12	127.40 ± 2.56^a^	84.40 ± 2.83^b^	75.80 ± 3.26^b^	74.80 ± 1.59^b^	18.80 ± 1.77^ab^
**Triglycerides (mg dL** ^-1^ **)**	52.20 ± 3.92	109.80 ± 11.12^a^	47.00 ± 3.16^b^	43.40 ± 2.97^b^	22.40 ± 2.90^ab^	11.20 ± 1.46^ab^

HCD: high cholesterol diet, HCD+L: low dose (100 mg kg^-1^) of *C. dactylon*, HCD+M: medium dose (200 mg kg^-1^) of *C. dactylon*, HCD+H: high dose (400 mg kg^-1^) of *C. dactylon,* and HCD+STN: atorvastatin (10 mg kg^-1^) group.

ab indicate significant difference compared to control and HCD groups, respectively at* p* < 0.05.

## Discussion

There are plenty of evidence that hyperlipidemia, inflammatory reactions and oxidative stress contribute to the development of atherosclerosis.^26^ The extract of *C. dactylon* has been used for treatment of diabetes mellitus and management of hyperlipidemia.^20^^,^^27^ However, the effects of this plant in the pathogenesis and development of atherosclerosis have not been investigated. In this study, we elucidated the ameliorative effects of *C. dactylon *extract on the pathogenesis of atherosclerosis in HCD rats.

Microscopical examinations of aortic tissue sections showed the lesions involve intimal and medial layers in HCD group. Atherosclerotic plaques initially develop as intimal precursor lesions at sites which are predisposed to lesion formation.^8^ Researchers have classified athero-sclerotic lesions into six categories based on light microscopical morphology. They have classified type I and II as fatty streak precursor type lesions. Type III has been considered as a transition stage lesion leading to type IV which is the classical atheroma. Type V is the complicated plaque with features of fibrosis and calcification and type VI is the full blown complicated lesion with surface erosions, fissures, plaque hematomas and surface thrombi with or without plaque rupture.^28^^,^^29^ Rats have been known as cholesterol resistant laboratory animals .^30^ To overcome this tolerance, along with HCD, we inhibit the secretion of thyroid hormones by PTU. Since thyroid hormone directly promotes cholesterol metabolism by liver, PTU induces high blood levels of cholesterol.^24^

Vascular fibrosis observed in microscopical examinations of coronary artery characterized by lumen diameter reduction and arterial wall thickness can be attributed to excessive deposition of ECM. According to previous report, vascular fibrosis involves VSMC proliferation, ECM accumulation and inhibition of matrix degradation.^31^ Vascular smooth muscle cells have important functions in normal physiology; however, in the microenvironment of injured artery wall, VSMC regulate development and growth of plaque. These cells in the plaque are mainly derived from tunica media, although normal intima has some VSMC. Other possible sources of VSMC are adventitia and bone marrow derived precursor cells in the circulation.^32^ Recently, *in vivo* studies have shown that mature medial VSMC may become activated and transdifferentiate to macrophage-like cells.^33^In order to identify proliferating cells (e.g., VSMC) within developing early atherosclerotic lesions, aorta and coronary arteries of HCD and treated groups were analyzed with PCNA monoclonal antibody technique. Negative immunoreactivity was revealed in the vascular wall of control group. Instead, proliferative index of VSMC was higher in HCD group. Our study showed that high dose of *C. dactylon* extract inhibits cellular proliferation in HCD arteries. The mechanism by which *C. dactylon* can reduce VSMC proliferation and atherosclerosis in injured arteries is probably at least in part related to its antioxidant and anti-inflammatory properties. Based on previous studies, hypercholesterolemia, increased circulating levels of oxidized LDL, can promote fibrosis and VSMC proliferation by stimulation of expression and synthesis of TGF-beta.^34^^-^^36^ In this study, vascular wall lesions were microscopically observed in rats fed HCD (higher egg yolk). This finding is in agreement with previous studies indicating that higher egg yolk consumption is associated with an increased prevalence of subclinical coronary atherosclerosis and carotid plaque.^37^^-^^41^In line with that, early stage of egg consumption related coronary atherosclerosis may be partially attributed to the dietary cholesterol in the yolk. Several mechanisms may explain the association between egg yolk consumption and coronary atherosclerosis. Early studies have mainly focused on the link between dietary cholesterol, serum cholesterol and cardiovascular diseases.^38^ In this study, we found some abnormalities in routine lipid profiles including increase of TC, triglycerides and LDL to HDL ratio. These findings indicated the association between egg yolk consumption and subclinical coronary atherosclerosis. Egg yolk consumption may also increase the risk of diabetes as a strong risk factor for atherosclerosis.^42^^,^^43^ Several studies have shown that diets enriched with egg yolk result in elevated plasma glucose.^44^

Previous studies have shown that infiltration of LDL in the arterial intima initiates an inflammatory response in the aorta wall and other large or medium-sized arteries.^8^ Oxidation of LDL in the arterial wall of all vertebrates is thought to be a very important step in atherogenesis.^8^^,^^45^ Thereby, reducing of serum level of LDL could be a preventive step in the development of atherosclerosis. We have observed that ethanolic extract of *C. dactylon *dose-dependently decreases blood cholesterol and LDL level in HCD rats. It was assumed that prevention of athero-sclerosis with antioxidants can be resulted from their ability to protect LDL from oxidation and lower serum cholesterol.^46^ It has been suggested that hypercholesterolemia diminishes antioxidant defense capacity and whereby elevates lipid peroxide contents^47^ confirming our findings. A decrease in lipid peroxidation leads to a reduction of atherosclerosis caused by hypercholesterolemia.^47^ The antioxidant potential of *C. dactylon *has shown greater *in vitro* ability based on estimating non-enzymatic hemoglobin glycosylation.^16^ Hypertriglyceridemia itself is an independent risk factor and can accelerate the development of atherosclerosis.^48^ Most of medications do not decrease TG levels, however, our results showed that *C. dactylon* not only reduces the lipid levels but also lowers the plasma level of TG. The TG accumulation caused by dietary cholesterol may be contributed to the reduction of fatty acid beta oxidation and the preference of cholesterol ester to afflux to LDL during the onset of biosynthesis and secretion of LDL.^49^ The significant decline in the serum TG concentration observed in the *C. dactylon* extract-treated rats supports the cardiovascular protective effects of this extract. The mechanism by which *C. dactylon* extract lowered serum TG concentration could be either through very low-density lipoprotein (VLDL) synthesis reduction, by channeling VLDL through pathways other than to LDL, or an increase in lipoprotein lipase activity.^21^

It can be concluded that ethanolic extract of *C. dactylon* lowers LDL and prevents from early atherosclerotic changes in heart vessel wall and aorta.
